# Supplementary material for: A Critical View of the Application of the APEX Software (Aqueous Photochemistry of Environmentally-Occurring Xenobiotics) to Predict Photoreaction Kinetics in Surface Freshwaters
Source: Molecules. 2019 Dec 18;25(1):9. doi: 10.3390/molecules25010009 (PMC7017383; doi:10.3390/molecules25010009)
Supplement: Supplementary file 1 [file molecules-25-00009-s001.zip › 3.2.4_gcc-4.4.0.zip!/3.2.4_gcc-4.4.0/3.2.4_gcc-4.4.0/doc/octave/html/interpreter/Errors.html]

Errors - Untitled


Next: Executable Octave Programs,
Previous: Command Line Editing,
Up: Getting Started


---

### 2.5 How Octave Reports Errors

Octave reports two kinds of errors for invalid programs.

A parse error occurs if Octave cannot understand something you
have typed. For example, if you misspell a keyword,

```
     octave:13> function y = f (x) y = x***2; endfunction
```

Octave will respond immediately with a message like this:

```
     parse error:
     
       syntax error
     
     >>> function y = f (x) y = x***2; endfunction
                                   ^
```

For most parse errors, Octave uses a caret (^) to mark the point
on the line where it was unable to make sense of your input. In this
case, Octave generated an error message because the keyword for
exponentiation (`**`) was misspelled. It marked the error at the
third \* because the code leading up to this was correct but the final
\* was not understood.

Another class of error message occurs at evaluation time. These
errors are called run-time errors, or sometimes
evaluation errors, because they occur when your program is being
run, or evaluated. For example, if after correcting the
mistake in the previous function definition, you type

```
     octave:13> f ()
```

Octave will respond with

```
     error: `x' undefined near line 1 column 24
     error: called from:
     error:   f at line 1, column 22
```

This error message has several parts, and gives quite a bit of
information to help you locate the source of the error. The messages
are generated from the point of the innermost error, and provide a
traceback of enclosing expressions and function calls.

In the example above, the first line indicates that a variable named
x was found to be undefined near line 1 and column 24 of some
function or expression. For errors occurring within functions, lines
are counted from the beginning of the file containing the function
definition. For errors occurring outside of an enclosing function,
the line number indicates the input line number, which is usually displayed
in the primary prompt string.

The second and third lines of the error message indicate that the error
occurred within the function `f`. If the function `f` had been
called from within another function, for example, `g`, the list of
errors would have ended with one more line:

```
     error:   g at line 1, column 17
```

These lists of function calls make it fairly easy to trace the
path your program took before the error occurred, and to correct the
error before trying again.
